# The prevalence of neural tube defects in eastern Ethiopia: A population-based surveillance

**DOI:** 10.1371/journal.pgph.0007059

**Published:** 2026-08-18

**Authors:** Helina Heluf, Hannah Blencowe, Kedir Teji Roba, Samrawit Abebaw, Merga Dheresa, Gezahegn Mengesha, Mohammed Jemal Mohammed, Mohammed Abdulkadir Omere, Faisel Abdi Hassen, Tadesse Dufera Tadesse, Yenenesh Tilahun, Yunus Edris, Tadesse Gure, Nega Assefa, Lola Madrid

**Affiliations:** 1 Department of Infectious Disease Epidemiology and International Health, London School of Hygiene & Tropical Medicine, London, United Kingdom; 2 College of Health and Medical Sciences, Haramaya University, Harar, Ethiopia; PLOS: Public Library of Science, UNITED STATES OF AMERICA

## Abstract

Neural tube defects (NTDs) are congenital anomalies associated with stillbirth, morbidity, disability, and premature death. The absence of population-level data in many settings hinders the planning of effective prevention and intervention strategies. This study aims to determine the prevalence of NTDs in eastern Ethiopia through a population-based surveillance conducted at Hararghe health and demographic surveillance system’s (HDSS) sites, eastern Ethiopia, from June 1, 2023, to May 31, 2024. All spontaneous pregnancy losses, children, and adults were eligible for inclusion. A paediatrician confirmed NTD diagnosis and classification. Birth prevalence and the prevalence in the population overall and by sex, age, residence, and NTD types were computed using HDSS and pregnancy surveillance denominators. Overall, 126 NTD cases were enrolled, yielding a birth prevalence of 98.7 (95% CI: 79.8-120.6) per 10,000 births. The prevalence of NTDs in the population was 1.8 (95% CI: 1.4-2.3) per 10,000 population. Prevalence varied by site, being highest in Kersa (birth prevalence: 140.6 (95% CI: 106.4-181.3) per 10,000 births; prevalence in the population: 2.2 (95% CI: 1.5-3.0) per 10,000 population) and in rural areas (birth prevalence: 128.7 (95% CI: 103.9-157.6) per 10,000 births; prevalence in the population: 1.9 (95% CI: 1.4-2.5) per 10,000 population). Spina bifida accounts for 51.6% of cases (birth prevalence: 49.3 (95% CI: 36.3-65.6) per 10,000 births; prevalence in the population: 1.5 (95% CI: 1.1-1.9) per 10,000 population). Of the 126 enrolled, 78 (62%) died during the study period (68 in-utero demises and 10 child deaths). High rates of stillbirths and low survival among affected live births resulted in a low population NTD prevalence contrasting with the high birth prevalence in the study area, particularly in rural areas. Comprehensive preventive strategies, including dietary diversification, preconception counselling, early screening and follow-up, folic acid supplementation, and mandatory folic acid fortification, to reduce NTDs burden, disability, and mortality.

## Introduction

Neural tube defects (NTDs) are a group of severe congenital anomalies of the central nervous system characterised by the incomplete closure of the embryonic neural tube within 20–28 days after conception [1–3]. Among the different types of NTDs, anencephaly and spina bifida are the most common, but craniorachischisis, encephalocele, and iniencephaly are also prevalent in high-burden areas [3–5]. NTDs are associated with inadequate folate status at the time of pregnancy [2,6] and other factors affecting folate metabolism, such as genetic or environmental factors, obesity, diabetes mellitus, and cigarette smoking [7–12].

There is a wide variation in the prevalence of NTDs worldwide, being higher in areas lacking mandatory food fortification programs [13,14]. Previous research on NTDs has primarily focused on birth prevalence. Based on population-based data in 2015, it was estimated that NTDs affected approximately 260,100 births globally, with an overall birth prevalence of 18.6 per 10,000 live births (95% CI: 15.3–23.0) and 14.2 per 10,000 live births (95% CI: 8.0–24.0) in Sub-Saharan Africa [14]. Among the regions in Africa, the highest prevalence of NTDs was reported in eastern Africa, with a birth prevalence of 111.1 per 10,000 births (95% CI: 91.85–130.42) [15].

However, reliable population-level data on the prevalence of NTDs are scarce in countries that lack robust birth defect surveillance systems, such as Ethiopia [16,17]. In this country, a systematic review and meta-analysis of hospital-based data estimated a pooled birth prevalence of 63.3/10,000 births (95% CI: 50.93-75.67), with the highest birth prevalence of 131/10,000 births (95% CI, 113–150.6) recorded in Tigray, northern Ethiopia [18,19]. Those hospital-based studies may overestimate NTDs prevalence amongst livebirths, as hospitals are more likely to attract complicated cases such as NTDs, but can also underestimate the actual burden, as they often exclude stillbirths and abortions, which may account for 50% of NTD cases [14].

Although literature on causes of death attributed to NTDs is scarce, an ongoing Child Health and Mortality Prevention Surveillance (CHAMPS) conducted in several countries in Sub-Saharan Africa and South Asia to ascertain causes of death in stillbirths and children aged <5 years found an adjusted mortality rate attributed to NTDs of 104.0/10,000 births (95% CI: 94·3–116·4) in its site in eastern Ethiopia. This was 4–23 times higher than other sites where CHAMPS operates [20].

Untreated NTDs are commonly fatal in utero or early life. While surgical repair improves survival, most survivors experience persistent motor, sensory, and neurological impairments [17,21–23]. Understanding the burden of NTDs will guide investments and health policies to prevent and improve care for affected individuals. This study aims to determine the birth prevalence of NTDs and the prevalence in the population across three health and demographic surveillance systems (HDSS) in eastern Ethiopia.

## Methods

### Study design, area and period

A population-based surveillance approach was used to estimate birth prevalence (including stillbirths and livebirths) and the prevalence of NTDs in the Hararghe HDSS, which operates in the Harar urban setting and in the rural settings of Haramaya and Kersa. The study was carried out from June 1, 2023, to May 31, 2024. The Harar HDSS comprises a total population of 53,684, including 16,765 women of reproductive age (WRA). Briefly, Kersa and Haramaya HDSS are located in the Oromia Regional State, eastern Ethiopia. Kersa HDSS has a total population of 148,996 with 35,855 WRA, while Haramaya HDSS has a total population of 120,377, with 28,248 WRA [24,25].

Hiwot Fana Comprehensive Specialised Hospital is one of the referral hospitals in the country, located in the Harari region with a catchment population of 5.8 million, including Harar, Haramaya, and Kersa HDSS sites. Details of Kersa and Haramaya HDSS have been published elsewhere [24,25].

### Population

The study population encompassed all foetuses in-utero, children and adults without any age restriction, currently alive or deceased. Cases identified at the time of death were classified into three categories based on gestational age and birth status: miscarriages (spontaneous pregnancy losses occurring before 28 weeks of gestation, with no lower gestational age limit); stillbirths (foetal deaths occurring at 28 weeks of gestation or later); and deaths (cases where the baby was born alive but subsequently died). This classification system was applied within the Hararghe HDSS in eastern Ethiopia. NTD cases were defined as those diagnosed with spina bifida, craniorachischisis, anencephaly, encephalocele, and iniencephaly. The prevalence of NTDs in the population was determined using data from the whole population of the Hararghe HDSS, collected during routine HDSS rounds. Birth prevalence was calculated using routine pregnancy surveillance data from the HDSS sites.

### Sampling procedure and techniques

**Fig 1:**
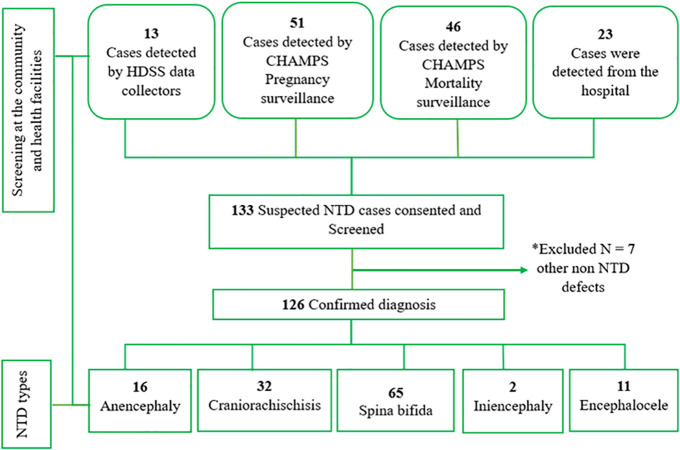
Flow diagram of neural tube defect cases identification from June, 2023 – May, 2024, eastern Ethiopia. *7 excluded includes: 5 lipomas, 1 dermoid cyst, and 1 macerated stillbirth without evidence of NTD.

Study participants with suspected NTDs were identified by staff working in various ongoing surveillance systems within the catchment area, including HDSS data collectors during their routine home visits and clinical staff involved in the ongoing pregnancy, mortality, and clinical surveillance systems. The pathways for this case identification flow are detailed in Fig 1.

HDSS data collectors and hospital staff detected liveborn cases, CHAMPS pregnancy surveillance detected neonates, stillbirths, miscarriages, and cases in utero, and CHAMPS mortality surveillance detected dead cases.

HDSS data collectors conduct two annual rounds to gather demographic data, including deaths and birth outcomes. During these routine visits, they identified NTD cases using drawings of different types of NTDs. The pregnancy surveillance enrols women as early as possible in their pregnancy through health extension workers, female community representatives with established community trust, HDSS data collectors, and health professionals. The first visit aligns with routine antenatal care (ANC), though data collection for CHAMPS pregnancy surveillance is conducted independently. During the study period, suspected pregnancies were confirmed by urine testing for early gestational ages or ultrasound. Gestational age was primarily determined through ultrasound examination, with last menstrual period used as an alternative when ultrasound was unavailable. The median gestational age at enrolment was 24 weeks. Women were followed through three scheduled study visits for data collection: at enrolment as soon as the pregnancy was notified, at delivery, and at day 42 postpartum. This assessment encompasses both home and institutional deliveries. Mortality surveillance is part of CHAMPS and investigates the causes of stillbirth and child death through integrated post-mortem sampling, clinical records, photographic evidence, and verbal autopsy. The mortality surveillance identified NTDs who were stillbirths or child deaths through both community members and health professionals, similar to the identification process for the pregnancy surveillance [26]. NTD-affected individuals from Hiwot Fana Comprehensive Specialised Hospital residing within Harar, Haramaya, and Kersa HDSS were identified and enrolled during daily rounds in the neonatal and paediatric wards through clinical surveillance.

All identified cases were linked to specific study nurses. Paediatricians and obstetricians trained data collectors and study nurses in NTD case identification using the Centres for Disease Control and Prevention and World Health Organisation (CDC/WHO) surveillance manual [27]. Study nurses approached caregivers of minors and other participants to explain the study and obtain signed informed consent, which included consent for case photographs. A pediatrician then reviewed each case using available information, medical records, and photographs to confirm the NTD diagnosis and classify it by type. Following the identification of cases through the integrated surveillance system described above, the cases and denominators were partitioned into two distinct groups for the study estimates:

**Birth prevalence**: cases were selected by isolating only those NTD diagnoses that resulted in a livebirth or stillbirth during the one-year study period. The denominators for this calculation were the total counts of all births (livebirths and stillbirths) identified by the pregnancy surveillance during the same timeframe (S1 Text).

**Prevalence of NTDs in the population**: the sampling included all liveborn individuals with NTDs identified through surveillance systems, regardless of their birth date. The denominator for this estimate was the total resident population of the Hararghe HDSS at the time of the study, as determined from demographic data collected during routine HDSS rounds (S1 Text).

### Data analysis

Data were checked for completeness, cleaned and exported to STATA version 18 for analysis. Descriptive statistics, including frequencies and percentages, were used to summarise the prevalence of NTDs, sociodemographic characteristics of the study participants, types of NTDs, and pregnancy outcomes. Birth prevalence was calculated for the overall study population in Hararghe HDSS, by sex (male and female), by residence (urban and rural), and for each site in the HDSS individually. With the study period defined as birth year, birth prevalence was calculated by dividing the number of NTD-affected births by the total number of births overall (livebirths and stillbirths) and in each respective group using matched numerators and denominators from the same period. The birth prevalence for each NTD type was also calculated by dividing the case count for each type by the total number of births. Stillbirth NTD prevalence was calculated by dividing the total number of NTD-affected stillbirths by the total number of births (livebirths and stillbirths). Livebirth NTD prevalence was calculated using the number of affected livebirths as the numerator and the total number of livebirths as the denominator. Miscarriages were excluded from the birth prevalence calculation because early miscarriages are difficult to capture as they mostly occur at home, and differentiating those with and without NTD is challenging at early gestational ages. To avoid neglecting this population, NTD prevalence among miscarriages was calculated separately, by dividing the number of miscarriages with NTDs by the total number of pregnancies. The prevalence of NTDs in the population was calculated by dividing the total number of living individuals with NTDs identified during the study period by the total population of the HDSS. Prevalence was stratified by age group (neonates, 28 days to <5 years, 5–18 years, and >18 years), sex, residence (urban and rural), and individual HDSS site, using the respective population denominators. The prevalence of each specific type of NTD in the population was also calculated by dividing the number of individuals living with the specific NTD by the total population. Pearson chi square analysis was conducted to assess for a statistically significant difference in prevalence across different categories of sex, HDSS sites, residence (urban or rural), and types of NTD. All statistical tests were two sided, and a p-value of <0.05 was considered statistically significant. Kaplan–Meier survival analysis was conducted in three stages. First, across all pregnancies in the surveillance population, we compared the timing of in-utero foetal demise between NTD-affected and unaffected pregnancies. Second, among all in-utero foetal demises, we evaluated the time to in-utero foetal demise specifically for NTD-affected pregnancies resulting in in-utero foetal demise and compared these patterns with foetal loss among unaffected pregnancies detected through the same pregnancy surveillance. Third, we analysed the time-to-death among all liveborn infants, both NTD-affected and unaffected, identified through pregnancy surveillance during the study period. For this Kaplan-Meier analysis, we restricted the time-to-event assessment to 42 days postpartum, reflecting the pregnancy surveillance follow-up period. The log-rank test was employed to evaluate the statistical significance of the differences between the survival distribution, with significance maintained at p < 0.05. To assess the independent association between NTDs and mortality while accounting for potential confounding, we performed multivariable Cox proportional hazards regression. Covariates included in the model were sex and HDSS site, as preliminary stratified log-rank analysis indicated that they significantly influenced the mortality hazard. Participants who did not experience the event of interest or were lost to follow-up were censored at their last day of contact or at the end of the follow-up period.

### Ethical consideration

This study received ethical clearance from the Institutional Health Research Ethics Review Committee (IHRERC) of Haramaya University (IHRERC/188/2024) and the London School of Hygiene and Tropical Medicine (31216). The study was conducted in accordance with the ethical principles of the Declaration of Helsinki for medical research involving human subjects. Official letter of cooperation was submitted to the respective district offices and health institutions prior to data collection. We obtained a written informed voluntary consent from all study participants (parents/ guardians for minors), and confidentiality was maintained throughout the process.

## Results

From June 1, 2023, to May 31, 2024, 133 suspected cases were screened, of which 126 were confirmed as NTD. All confirmed cases agreed to participate in the study (Fig 1). Out of the 126 NTD cases enrolled, 56 (44.4%) were live births, 46 (36.5%) were stillbirths, 13 (10.3%) were alive in utero, and 11 (8.7%) were miscarriages. For the birth prevalence estimate, 21 of 56 initial liveborn cases were excluded as their date of birth was before the study period, and 11 were miscarriages, resulting in a final sample of 94. Of the 13 initially identified as alive in utero, 11 were stillborn, and 2 were born alive during the study period. For the prevalence of NTDs in the population, all 58 (56 at recruitment + two from alive in utero) liveborn cases (28 neonates, 24 children aged 28 days to <5 years, six children aged 5–18 years) were included (S1 Text).

### Sociodemographic and clinical characteristics

Of the 126 enrolled NTD cases, 70 (55.6%) were males, 119 (94.4%) resided in rural areas, and 73 (57.9%) were from the Kersa HDDS site. Overall, 56 (44.4%) were born alive at recruitment, of whom 26 (46.4%) were neonates. Spina bifida was the most common NTD diagnosed, accounting for over half of all cases (65, 51.6%). Additionally, 32 (25.4%) and 16 (12.7%) of the cases were craniorachischisis and anencephaly, respectively (Table 1).

**Table 1:** Sociodemographic and clinical characteristics of neural tube defect cases from June 1, 2023 – May 31, 2024, eastern Ethiopia (n = 126).

Variables	Frequency	Percentage (%)
**HDSS site**		
Harar	7	5.6
Haramaya	46	36.5
Kersa	73	57.9
**Residence**		
Urban	7	5.6
Rural	119	94.4
**Sex of the study participant**		
Male	70	55.6
Female	56	44.4
**Vital status at recruitment**		
Alive in-utero	13	10.3
Miscarriage (<28 weeks)	11	8.7
Stillbirth (>28 weeks)	46	36.5
Alive	56	44.4
**Age at recruitment (livebirths only)**		
Neonate (0 – 27 days)	26	46.4
28 days - < 5 years	24	42.9
5-18 years	6	10.7
Adult	0	0.0
**NTD type**		
Spina bifida	65	51.6
Anencephaly	16	12.7
Craniorachischisis	32	25.4
Encephalocele	11	8.7
Iniencephaly	2	1.6

NTD: Neural tube defects, HDSS: Health and demographic surveillance system

### Prevalence of NTD

The birth prevalence of NTDs was 98.7 (95% CI: 79.8-120.6) per 10,000 total births overall, with significant geographical variation, ranging from 7.9 (95% CI: 0.9–28.5) in urban areas to 128 (95% CI: 103.9-157.6) in rural areas. Among the 266 stillbirths recorded across the three HDSS sites during our study period, 57 (21.4%) were associated with NTD, with a birth prevalence of 59.8 per 10,000 births (95% CI: 45.4-77.5). The birth prevalence in males was 102.7 (95% CI: 76.7-134.4) per 10,000 total male births (Table 2).

**Table 2:** Birth prevalence of neural tube defects from June 1, 2023 – May 31, 2024, eastern Ethiopia (n = 94).

Population	NTD Cases (n (%))	Total birth (denominator)	Prevalence per 10,000 total births (95% CI)	p-value
**Birth prevalence**	94	9,524^a^	98.7 (79.8-120.6)	0.058
Stillbirth	57 (60.6)	9,524^a^	59.8 (45.4-77.5)	
Livebirth	37 (39.4)	9192^b^	40.3 (28.4-55.4)	
**Sex**				0.689
Males	52 (55.3)	5064^c^	102.7 (76.7-134.4)	
Female	42 (44.7)	4442^d^	94.6 (68.2-127.6)	
**HDSS site**				**<0.01**
Harar	2 (2.1)	2534^e^	7.9 (0.9-28.5)	
Haramaya	35 (37.2)	3085^f^	113.5 (79.1-157.4)	
Kersa	57 (60.7)	4064^g^	140.6 (106.4-181.3)	
**Residence**				**<0.01**
Urban	2 (2.1)	2534^e^	7.9 (0.9-28.5)	
Rural	92 (97.9)	7149^h^	128.7 (103.9-157.6)	
**Types of NTD**				**<0.01**
Spina bifida	47 (50.0)	9,524^a^	49.3 (36.3-65.6)	
Anencephaly	14 (14.9)	9,524 ^a^	14.7 (8.0-24.6)	
Craniorachischisis	23 (24.5)	9,524 ^a^	24.1 (15.3-36.2)	
Encephalocele	8 (8.5)	9,524 ^a^	8.4 (3.6-16.5)	
Iniencephaly	2 (2.1)	9,524 ^a^	2.1 (0.3-7.6)	

a :Total birth(livebirth + stillbirth), ^b^: Total livebirth, ^c^: Total male birth (livebirth + stillbirth), ^d^: Total female birth (livebirth + stillbirth), ^e^: Total birth in Harar (livebirth + stillbirth), ^f^: Total birth in Haramaya (livebirth + stillbirth), ^g^: Total birth in Kersa (livebirth + stillbirth), ^h^: Total birth in rural (Haramaya and Kersa (livebirth + stillbirth))

*miscarriages and births outside the study period were excluded from birth prevalence calculations, explaining discrepancies between Table 1 and Table 2 frequencies.

NTD: Neural tube defects, HDSS: Health and demographic surveillance system

Additionally, 11 (7.8%) of 141 recorded miscarriages were confirmed NTD cases, a prevalence of 11.38 (95% CI: 5.7-20.4) per 10,000 pregnancies.

Overall, 58 NTD-affected individuals were liveborn, resulting in a prevalence of NTDs in the population of 1.8 (95% CI: 1.4-2.3) and 1.9 (95% CI: 1.4-2.5) per 10,000 population in rural areas. The prevalence of NTDs in the population among males was 2.1 (95% CI: 1.4-2.9) per 10,000 male population, and in Kersa, it was 2.2 (95% CI: 1.5-3.0) per 10,000 population. Spina bifida was the most prevalent NTD type (1.5 (95% CI: 1.1-1.9) per 10,000 population (Table 3).

**Table 3:** Prevalence of neural tube defects in the population from June 1, 2023 – May 31, 2024, eastern Ethiopia (n = 58).

Population	Alive NTD Cases (n (%))	Total population (denominator)	Prevalence per 10,000 population (95% CI)	p-value
**Prevalence of NTDs in the population**	58	323,058 ^a^	1.8 (1.4-2.3)	**<0.01**
Neonates (0–27 days)	28 (48.2)	9,192^b^	30.5 (20.3-43.9)	
28 days - < 5 years	24 (25.5)	39,428 ^c^	6.1 (3.9-9.1)	
Children 5 – 18 years	6 (6.3)	118,020^d^	0.5 (0.2-1.1)	
**Sex**				0.223
Male	34 (58.6)	163,526^e^	2.1 (1.4-2.9)	
Female	24 (25.5)	159,532^f^	1.5 (0.9-2.2)	
**HDSS site**				**<0.01**
Harar HDSS	6 (10.3)	53,684^g^	1.1 (0.4-2.4)	
Haramaya HDSS	20 (34.5)	120,377^h^	1.7 (1.0-2.6)	
Kersa HDSS	32 (55.2)	148,996^i^	2.2 (1.5-3.0)	
**Residence**				0.199
Urban	6 (10.3)	53,684^g^	1.1 (0.4-2.4)	
Rural	52 (89.7)	269,373^j^	1.9 (1.4-2.5)	
**NTD type**				**<0.01**
Spina bifida	48 (82.8)	323,058^a^	1.5 (1.1-1.9)	
Encephalocele	10 (17.2)	323,058^a^	0.3 (0.1-0.6)	

^a^:Total population, ^b^: Total livebirth, ^c^: Total population between 28 days - < 5 years, ^d^: Total population between 5 and 18 years, ^e^:Total male population, ^f^:Total female population, ^g^: Total population in Harar, ^h^: Total population in Haramaya, ^i^:Total population in Kersa, ^j^: Total population in rural (Haramaya and Kersa)

NTD: Neural tube defects, HDSS: Health and demographic surveillance system

### Survival status of NTD cases

**Fig 2:**
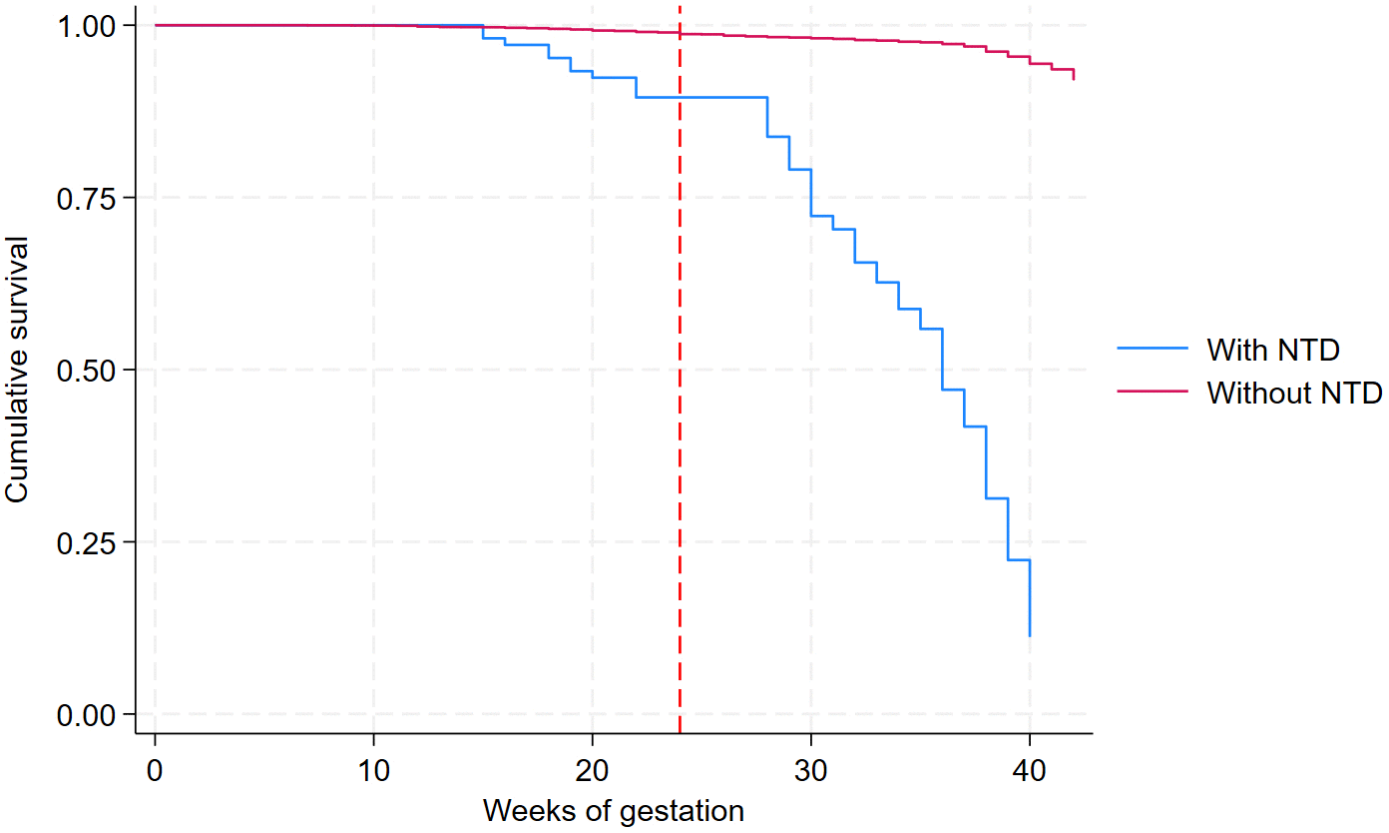
Kaplan-Meier survival estimates for NTD-affected and non-NTD pregnancies from June 1, 2023 – May 31, 2024, eastern Ethiopia.

During the study period, a total of 9,665 pregnancy outcomes were recorded. Pregnancies without NTD accounted for 9560 outcomes, distributed as 9221 (96.5%) livebirths, 209 (2.2) stillbirths, and 130 (1.3) miscarriages. The remaining 105 pregnancies with NTDs resulted in 37 (35.2) livebirths, 57 (54.3) stillbirths, and 11 (10.5) miscarriages (S1 Table). The Kaplan-Meier survival analysis of all pregnancy outcomes (with and without NTDs) demonstrates a significantly reduced survival for pregnancies with NTD compared to those without (Log Rank p < 0.01). The survival probability for the non-affected group remained near 1.00. In contrast, the affected group’s survival curve showed a sharp decline, particularly after 30 weeks of gestation, dropping to approximately 25% by around 40 weeks of gestation. The mean gestational age at enrolment into pregnancy surveillance was 24 weeks for our study period. A red dotted line is placed on the X-axis of the Kaplan-Meier curve at 24 weeks, which indicates the gestational age from which reliable event monitoring and follow-up commenced (Fig 2). Additionally, this association remained highly significant when the analysis was stratified by sex (X^2^ (1) = 1490.6, *p* < 0.01) and by HDSS site (X^2^ (1) = 1934.8, *p* < 0.01). After adjusting for sex and HDSS site using cox proportional hazards regression, the hazard ratio (HR) for mortality in the NTD group was 0.017 (95% CI: 0.01-0.02; p < 0.01). Both sex (female) (HR:0.76; p < 0.01) and study site (HRs: 0.24 for Haramaya site and 0.13 for Kersa site; p < 0.01) were statistically significant independent predictors of mortality. The proportional hazard assumption was satisfied (p > 0.05).

**Fig 3:**
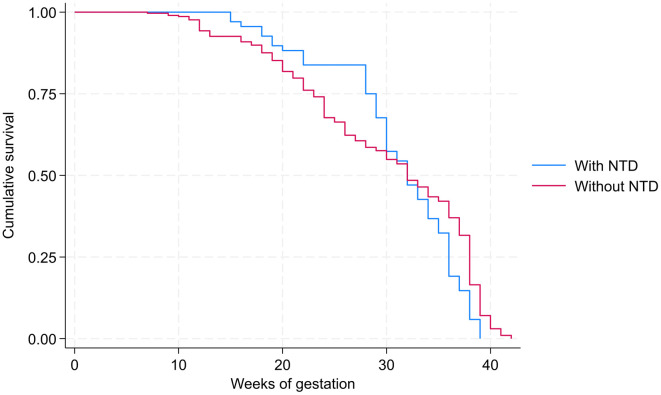
Time to foetal demise for NTD-affected and non-NTD pregnancies from June 1, 2023 – May 31, 2024, eastern Ethiopia.

Of the 126 NTD-affected cases recruited, 78 (61.9%) died before the end of the study period, including 68 in-utero demises and 10 child deaths (seven neonatal and three post-neonatal deaths) (S2 Table). The median week of gestation at in-utero demise for those with NTD (78 cases) was 32 weeks (interquartile range (IQR): 28–36 weeks) and for those without NTD (407 pregnancy losses), 32 weeks (IQR: 23–38 weeks). A dynamic pattern of pregnancy loss was observed across pregnancies with and without NTDs, characterised by an initial stable period followed by a gradual increase in loss. Notably, despite the overall similarity in temporal trends, a distinct plateau in the survival curve for pregnancies with NTDs occurred between 22 and 28 weeks of gestation. While cases showed a lower survival trend, a Log-rank (Mantel - cox) test indicates no statistically significant difference between those with and without NTD (P = 0.075) (Fig 3). However, when stratified by sex, the difference became highly significant (X^2^ (1) = 8.43, p < 0.01), indicating that sex is a significant source of variation in survival. Stratification by HDSS site attenuated the observed difference (X^2^ (1) = 1.16, p < 0.281). After adjusting for sex and HDSS site using cox proportional hazards regression, the HR for mortality in the NTD group was 0.746 (95% CI: 0.553-1.006; p = 0.054). neither sex nor HDSS site were statistically significant predictors of mortality in the final multivariable model, though the substantial change in the log-rank test upon stratification suggests sex is a key variable to account for when analysing survival in this population. The proportional hazards assumption was satisfied (p > 0.05).

**Fig 4:**
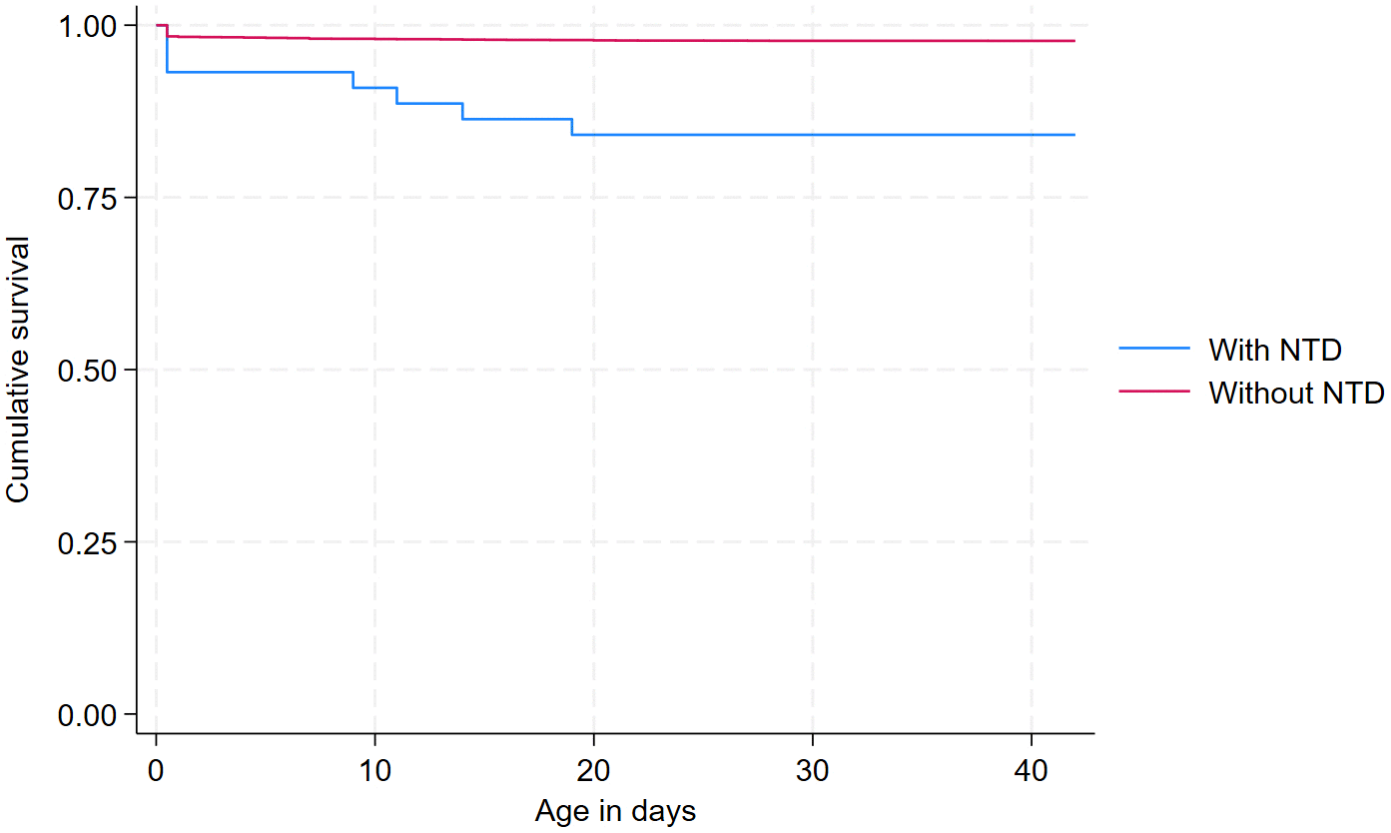
Kaplan-Meier survival estimates for NTD-affected and unaffected livebirths from June 1, 2023 – May 31, 2024, eastern Ethiopia.

From a total of 9192 live births in the study period, 215 died (seven with NTD and 208 without NTD) within 42 days postpartum (S3 Table). The estimated probability of survival to the end of the follow-up period (42 days) was markedly lower for newborns with NTD at approximately 0.83, compared to the group without NTD, which maintained a survival probability close to 1.00 throughout the observation period. The observed cumulative incidence of death was therefore substantially higher in the cohort with NTDs, with the majority of deaths occurring within the first 20 days postpartum. The Log-rank test confirms that the difference in survival between these groups is statistically significant (P < 0.001) (Fig 4). This association remained statistically significant and strengthened when the analysis was stratified by sex (X^2^ (1) = 39.2, p < 0.01) and by HDSS site (X^2^ (1) = 208.34, p < 0.01). After adjusting for sex and HDSS site using cox proportional hazards regression, the HR for mortality in the NTD group was 0.014 (95% CI: 0.005-0.043; p < 0.01). Both sex (HR: 1.416,p = 0.013) and HDSS site (HRs: 9.714 for Haramaya site and 13.785 for Kersa site; p < 0.01) were found to be statistically significant independent predictors of mortality. The proportional hazards assumption was satisfied (p < 0.05).

## Discussion

Our findings reveal a substantial prevalence of NTDs in this region of eastern Ethiopia, with a birth prevalence of 98.7 per 10,000 total births (livebirths and stillbirths), significantly higher than the global estimate of 18.6 per 10,000 live births [14]. While the global estimate primarily utilises livebirths as a denominator, our inclusion of stillbirths provides a more comprehensive capture of the birth prevalence in this high burden setting. Our prevalence of NTDs in the population of 1.8 per 10,000 population indicates a low survival among affected children. The burden was significantly higher in rural areas, affecting 1.3% of all births. These high prevalences underscore the critical need for effective prevention strategies in this region.

The observed high birth prevalence of NTDs is likely multifactorial. Differences in socioeconomic status, nutritional status, and geographical location likely contribute to the higher birth prevalence observed in this study compared to other African studies, where birth prevalence varies from 10 to 39.2 per 10,000 births [28–31] and compared to other regions of Ethiopia with birth prevalences ranging from 28.6 to 63.4 [32–35]. Observed differences may also reflect methodological disparities. For instance, our community-based prospective surveillance, which included routine ultrasound, likely detected more cases than the retrospective facility-based chart reviews used in other studies. In our study, almost half (48.2%) of NTD cases were home births and would likely have been missed using a facility-based approach. Additionally, mandatory folic acid fortification programs in some African countries could partly explain the lower NTD prevalence reported in those settings [36–39]. Global evidence confirms that mandatory folic acid fortification is a highly effective prevention strategy, with studies consistently demonstrating a substantial reduction in NTD prevalence following implementation [40]. Furthermore, the high birth prevalence in our study likely stems from limited early ANC and ultrasound screening, as only 10% of cases were identified prenatally, and detection often occurred late in gestation. This, coupled with low acceptance of elective termination for severe NTD types in the country, contrasts with regions where early ANC and termination access are widely accessible and acceptable. This could lead to lower reported birth prevalences, as their calculations often exclude terminated pregnancies [41]. Our observed birth prevalence is lower than some prior studies within Ethiopia (131–360 per 10,000 births) [18,42–44]. These hospital-based prevalence studies, particularly those conducted in tertiary or referral hospitals, may have overestimated population-level birth prevalence of NTDs. This is because NTD cases are referred from the community and lower-level facilities for surgical care, inflating the numerator, while the denominator remains limited to births occurring within the hospital, thereby artificially elevating reported prevalence. Consistent with this, our study’s population-based design, drawing on a large pregnancy surveillance cohort as a denominator, likely accounts for comparatively lower birth prevalence we observed, and may provide a more accurate reflection of the true burden of NTDs in the community.

Among the three HDSS sites, the birth prevalence was significantly higher in Kersa and Haramaya (140.6 (95% CI: 106.4-181.3) and 113.5 (95% CI: 79.1-157.4) per 10,000 births) than in Harar (p < 0.01). This may be attributed to the socioeconomic and environmental context of Kersa and Haramaya HDSS, characterised by a predominantly rural setting, low levels of education, limited access to healthcare settings, and potential nutritional deficiencies. Additionally, women’s dietary folate intake in Kersa is likely low, falling below the level necessary for preventing NTDs [45]. Our findings indicate no significant difference in the prevalence of NTDs between males and females.

The distribution of NTD sub-types at birth in our study showed spina bifida (49.1%) as the most common, aligning with patterns in other Ethiopian regions, Jimma and Amhara [32,43]. Conversely, other Ethiopian and African studies reported anencephaly as the most prevalent NTD (48.1–50%) [29,30,46]. A quarter (25.4%) of all NTD cases in our study were craniorachischisis, contrasting sharply with previous studies, where it is rarely reported and the most severe NTD type [34]. This discrepancy likely arises from our inclusion of pregnancy loss (miscarriages and stillbirths) and livebirths, providing a fuller picture of NTD prevalence. Additionally, paediatrician confirmation improved differentiation of craniorachischisis and anencephaly. Our birth prevalence of spina bifida of 49.3 per 10,000 births is consistent with Ethiopian studies from Tigray and Addis Ababa (64.4 and 51.0 cases per 10,000 births, respectively) [18,33], but it is higher than that reported in Hargeisa, Somaliland (3.6 per 10,000 births) [28]. Observed variations may arise from differential gene frequencies that affect genetic predisposition, differences in maternal folic acid levels due to diet and access to periconceptional folate intake [47] and varying environmental exposures to toxins. Additionally, our community-based design captures all births, including milder NTDs, unlike hospital-based studies, which, particularly in referral settings that often represent severe NTDs like anencephaly due to in utero referrals.

The prevalence of NTDs in the population among liveborn was 1.8 per 10,000 population. To our knowledge, this study provides the first assessment of the prevalence of NTDs in the population in Ethiopia. The low observed prevalence of NTDs in the population contrasts with a high birth prevalence, suggesting significant early mortality associated with NTDs, and limited survival into adulthood, as no affected adults were identified. Limited access to neurosurgical care until recently compounded by low awareness, stigma and cost, likely contributes to poor outcomes. Despite the presence of neurosurgical centre at the referral hospital in the catchment area since 2020, increasing awareness, and a surveillance system linking living individuals to neurosurgery, a quarter of all NTD-affected livebirths (2023–2024) in this study still died during the study period. Additionally, although the birth prevalence of NTDs was significantly higher in rural areas (p < 0.01), the prevalence of NTDs in the population shows minimal urban-rural difference. This could be attributed to better survival rates of live-born NTD cases in urban areas due to easier access to neurosurgery units, thus evening out the population prevalence figures.

All Kaplan–Meier analyses suggest that our study and pregnancy surveillance missed miscarriages before 10 weeks of gestation, underestimating early pregnancy loss. This likely reflects a limitation of surveillance systems in accurately capturing early pregnancies and foetal outcomes, highlighting the need for better methods to quantify early pregnancy loss and its causes, including birth defects. NTD dramatically reduces the likelihood of the foetus reaching full term, confirming the high intrauterine mortality risk. Among foetal demises, we found that NTD-related pregnancy loss remains relatively stable between 22 and 28 weeks of gestation and decline after 28 weeks suggests a threshold beyond which foetal compensatory mechanisms are no longer sufficient, leading to increased vulnerability and rapid demise. This deterioration is likely driven by NTD severity, often worsened by structural anomalies (cardiac, renal, and chromosomal), secondary complications (polyhydramnios, placental issues, and infection), and maternal conditions like uncontrolled diabetes [48,49]. In contrast, non-NTD foetal demise exhibits a more varied pattern across gestational ages, reflecting diverse underlying causes and pathways. Among livebirths, the pronounced decline in survival for infants with NTDs, primarily within the first 20 days postpartum, highlights the immediate, life-threatening nature of these complex congenital anomalies. This early mortality is often attributable to associated complications such as hydrocephalus, severe neurological impairment, or immediate post-surgical complications. Therefore, these results underscore the critical need for rapid, highly specialized neurosurgical and intensive postnatal care in the first few weeks of life to mitigate the high early mortality associated with NTDs.

A significant strength of this study is its community-based approach, which directly enhances the accuracy of the prevalence estimates. Furthermore, this report provides the first population-level prevalence data in Ethiopia before the full implementation of a food fortification program. As such, it will serve as a crucial baseline to assess the impact of the upcoming mandatory folic acid food fortification initiatives planned by the Ethiopian Ministry of Health. This research is further strengthened by the use of three established prospective surveillance systems within CHAMPS (mortality, clinical, and pregnancy). These systems utilise standardised data collection protocols and routine data audits, minimising the risk of NTD case under-reporting. Paediatrician-confirmed diagnoses against standardised case definitions further enhance the reliability of our findings. Furthermore, the use of continuous data from the HDSS and pregnancy surveillance, which undergo regular logic checks and a data cleaning cycle, provides robust denominators for our analysis. Despite its strengths, this research has several limitations. First, accurately identifying NTD-affected miscarriages in the first trimester was difficult, as these often occur before a diagnosis can be made or ultrasound screening is performed. Second, the total number of miscarriages was likely underestimated, which might consequently leads to an underestimation of the true burden of NTDs in the population. This ascertainment bias likely disproportionately affects rural communities compared to urban HDSS sites, given the challenges of lower health seeking behaviour and greater physical distance to healthcare facilities in rural area. However, because these cases were excluded from our birth prevalence calculations, the observed geographical variations in reported prevalence are not confounded by these differences in miscarriage ascertainment. Crucially, case ascertainment for stillbirths and livebirths remained robust and consistent across all sites due to uniform surveillance intensity. Third, reliance on gestational age at delivery or miscarriage, rather than foetal demise, introduces a potential, though likely small, imprecision in estimating the timing of adverse events. Fourth, the 42-day postpartum follow-up period mandated by the pregnancy surveillance protocol resulted in right-censored observations, preventing the determination of time to death for live born children beyond this window and likely underestimating the full mortality burden. Finally, given the small number of HDSS sites, formal multilevel modelling or cluster-robust variance estimation would be underpowered. We therefore utilized site stratified prevalence estimates. These findings should be interpreted with caution, as site specific variations may reflect both trues geographical differences and potential subtle variations in surveillance capture.

## Conclusions

Our findings reveal a high burden of NTDs in eastern Ethiopia, with marked rural disparities and substantial mortality among affected pregnancies, underscoring an urgent public health concern. As a population-based surveillance study conducted within an established HDSS, this work provides rare population-level evidence to guide prevention and intervention planning in a setting where such data have been lacking. These findings call for mandatory folic acid food fortification of a widely consumed staple food, strengthened preconception care and early ANC, and targeted community-based outreach in the highest-burden areas. Advanced care planning for affected pregnancies and sustained investment in cross-sectoral collaboration will be essential to reduce NTD-related mortality and disability in eastern Ethiopia. Given the high burden and mortality of NTDs, further research should prioritize evaluating the folate status of women of reproductive age and identifying specific environmental drivers, while longitudinal studies should explore the psychosocial and economic impacts on affected children and their families, to inform targeted interventions focusing on strengthening community-based surveillance to ensure the early identification of pregnancies, preconception care and comprehensive support of affected children and their parents.

## Supporting information

S1 Text Discrepancies in data presentation: a detailed explanation. (DOCX)

S2 Text STROBE checklist for cross-sectional study. (DOCX)

S1 Table Distribution of pregnancy outcomes by gestational age. (DOCX)

S2 Table Distribution of miscarriages and stillbirths by gestational age. (DOCX)

S3 Table Distribution of infant deaths within 42 days after birth. (DOCX)
