# Supplementary material for: The prevalence of neural tube defects in eastern Ethiopia: A population-based surveillance
Source: PLOS Glob Public Health. 2026 Aug 18;6(8):e0007059. doi: 10.1371/journal.pgph.0007059 (PMC13485017; doi:10.1371/journal.pgph.0007059)
Supplement: Supplementary file 1 [file pgph.0007059.s001.docx]

**Supporting Information**

**Discrepancies in data presentation: a detailed explanation**

This document explains the differences in the numerical data presented in the tables for sociodemographic and clinical characteristics, birth prevalence, and population based prevalence within the main manuscript. The primary distinction lies in the inclusion criteria for the prevalence calculations. Birth prevalence figures are based exclusively on individuals born during the designated study period. In contrast, population-based prevalence figures encompass all living cases at the time of the study, irrespective of their birth date, offering a measure of the total prevalence of neural tube defects within the defined population.

1. **Initial recruitment (126 cases)**

- 46 (36.5%) stillbirths
- 13 (10.3) alive in utero,
- 11 became stillbirths
- 2 livebirths
- 11 (8.7%) miscarriages
- 56 (44.4%) live births

2. **Filtering for birth prevalence estimate (94 cases)**

- **Excluded (32 cases)**
- 21 born out of the study period
- 11 were miscarriages

**Remaining 94 cases**

- **Live births in the study period = 37**
- 56 initial live births
- + 2 from alive in utero
- – 21 born outside the study period
- **Stillbirths in the study period = 57**
- 46 initial stillbirths
- + 11 from alive in utero

3. **Population based prevalence analysis (58)**

- 56 initially recruited alive
- + 2 from alive in utero

= 58 cases

- **Breakdown**
- 28 neonates (26 initially recruited + 2 from the alive in utero)
- 24 children aged 29 days to <5 years
- 6 aged 5 to18 years
