# Supplementary material for: The prevalence of neural tube defects in eastern Ethiopia: A population-based surveillance
Source: PLOS Glob Public Health. 2026 Aug 18;6(8):e0007059. doi: 10.1371/journal.pgph.0007059 (PMC13485017; doi:10.1371/journal.pgph.0007059)
Supplement: Supplementary file 3 [file pgph.0007059.s003.docx]

**Supporting information**

S1 Table: Distribution of pregnancy outcomes by gestational age for NTD affected and unaffected pregnancies.

| **Gestational age** | **Pregnancies with NTD** | | **Pregnancies without NTD** | |
| --- | --- | --- | --- | --- |
|  | **Stillbirth/ miscarriage** | **Alive** | **Stillbirth/ miscarriage** | **Alive** |
| < 24 weeks | 11 (16.2%) | 0 | 90 (26.5%) | 0 |
| 24-28 weeks | 6 (8.8%) | 0 | 62 (18.3%) | 8 (0.1%) |
| 29-37 weeks | 41(60.3%) | 11(29.7%) | 86 (25.4%) | 1386 (15.0%) |
| >37 weeks | 10 (14.7%) | 26 (70.3%) | 101 (29.8%) | 7827 (84.9%) |
| Total | 68 (100%) | 37 (100%) | 339 (100%) | 9221 (100%) |

We have included the total number of all pregnancy outcomes (live births and stillbirths/miscarriages) that occurred during the study period, categorized by gestational age and stratified by the presence or absence of NTD.
