# Supplementary material for: The prevalence of neural tube defects in eastern Ethiopia: A population-based surveillance
Source: PLOS Glob Public Health. 2026 Aug 18;6(8):e0007059. doi: 10.1371/journal.pgph.0007059 (PMC13485017; doi:10.1371/journal.pgph.0007059)
Supplement: Supplementary file 4 [file pgph.0007059.s004.docx]

**Supporting information**

S2 Table: Distribution of miscarriages and stillbirths by gestational age for NTD affected and unaffected pregnancies.

| **Gestational age** | **In-utero demise with NTD** | **In-utero demise without NTD** |
| --- | --- | --- |
| < 24 weeks | 11 (16.2%) | 90 (26.5%) |
| 24-28 weeks | 6 (8.8%) | 62 (18.3%) |
| 29-37 weeks | 41(60.3%) | 86 (25.4%) |
| >37 weeks | 10 (14.7%) | 101 (29.8%) |
| Total | 68 (100%) | 339 (100%) |

We have included in-utero foetal demises (stillbirths and miscarriages combined) that occurred during the study period, categorized by gestational age and stratified by the presence or absence of NTD.
