# Supplementary material for: The prevalence of neural tube defects in eastern Ethiopia: A population-based surveillance
Source: PLOS Glob Public Health. 2026 Aug 18;6(8):e0007059. doi: 10.1371/journal.pgph.0007059 (PMC13485017; doi:10.1371/journal.pgph.0007059)
Supplement: Supplementary file 5 [file pgph.0007059.s005.docx]

**Supporting information**

S3 Table: Distribution of infant deaths within 42 days after birth for NTD affected and unaffected livebirths.

| **Days after birth** | **Livebirths with NTD** | | **Livebirths without NTD** | |
| --- | --- | --- | --- | --- |
|  | **Death** | **alive** | **Death** | **alive** |
| 0 – 24hrs | 3 (42.9%) | 34 (91.9%) | 148 (71.2%) | 9037 (98%) |
| 1-6 days | 0 | 34 (91.9%) | 23 (11.1%) | 9050 (98.1%) |
| 7 – 27 days | 4 (57.1%) | 30 (81.1%) | 35 (16.8%) | 9015 (97.8%) |
| 28 – 42 days | 0 | 30 (81.1%) | 2 (0.9%) | 9013 (97.7%) |
| Total | 7 (100%) | 37 (100%) | 208 (100%) | 9221 (100%) |

We have included the timing of child deaths (post-live birth) within the 42-day follow-up period, categorized by days after birth and stratified by the presence or absence of NTD.
